# Consumer Fraud against Older Adults in Digital Society: Examining Victimization and Its Impact

**DOI:** 10.3390/ijerph20075404

**Published:** 2023-04-05

**Authors:** Steven Kemp, Nieves Erades Pérez

**Affiliations:** 1 Department of Public Law, University of Girona, 17003 Girona, Spain; 2 Department of Health Psychology, Miguel Hernández University of Elche, 03202 Elche, Spain

**Keywords:** buying scams, identity theft, monetary fraud, psychosocial impacts, senior citizens, elderly

## Abstract

The European population is aging, which means more people aged sixty-five and over are at risk of financial exploitation. However, there is a lack of consensus regarding whether older persons are at greater risk of fraud than younger counterparts due to physical, economic, and social factors or, rather, whether they are slightly protected from fraud in the digital era due to less frequent online activity. Moreover, little is known about the financial, emotional, psychological, and physical impacts of fraud experiences amongst older generations in digital society. We employ multilevel modelling on a sample of EU citizens (*n* = 26,735) to analyze these issues. The results show that, holding other factors constant, older adults are more likely to suffer fraud in general, but not fraud via online channels. Identity theft in which the offender attempts to trick the victim by impersonating a reputable organization is found to be particularly relevant for citizens aged sixty-five and above. Older persons are less likely to suffer a financial impact but more likely to experience anger, irritation, embarrassment, and negative impacts on their physical health from fraud in general as well as from online fraud. Many organizations aim to help protect older adults from financial crime and its impacts; thus, the results emphasize the need to understand particular fraud categories suffered by older generations and to design support programs that fully take into account the non-financial impacts of this crime.

## 1. Introduction

According to the Council of Europe Development Bank, population aging will be one of the clearest demographic trends in the 21st century [1]. For instance, rising life expectancy and low fertility rates mean the proportion of the European Union population aged sixty-five and above is predicted to increase from 20% in 2019 to 30% in 2070, with those aged over eighty expected to more than double [2]. These demographic changes will likely be accompanied by growth in the number of older people who suffer financial exploitation such as fraud [3,4,5]. In accordance with the European Commission [6], in the present article we understand older adults to be persons aged sixty-five and over. Various sources have already identified senior citizens as being particularly vulnerable to fraud [7,8], thereby necessitating improved understanding of the issue and the relevant prevention measures.

Despite the prevalence of fraud within the oldest demographic groups, it is still unclear whether they are at greater risk compared to other age groups. On the one hand, some research has found increased vulnerability due to a combination of physical, economic, and social factors [9,10,11,12]. Firstly, with regards to physical elements, a reduction in cognitive capacities due to deterioration of the prefrontal cortex has been linked to excessive credulousness and diminished financial decision making, thereby increasing vulnerability to fraud [4,12,13,14]. It is also commonly accepted that older generations are less technologically adept than their younger counterparts, and their lack of technological knowledge has been associated with increased risk of cyber fraud victimization [11]. Secondly, from an economic perspective, the wealth and assets of older people mean that they are attractive targets for fraudsters [3,15]. The greater personal wealth accumulated over a lifetime increases the risk of targeting in comparison to younger generations [16]. Finally, in terms of social factors, social isolation may make older persons easier to manipulate and may reduce their awareness of potential fraud risks [10,17,18,19]. On the other hand, it has been shown that fraud victimization now mainly occurs online [20], and senior citizens are believed to be at a lower risk of online financial victimization than other age groups because they are less digitally active [21]. This conclusion is supported by research showing that internet use and online purchasing are linked to greater levels of cyber fraud targeting [22,23,24,25].

However, internet usage in older adults is also rising. According to Eurostat, in the decade between 2010 and 2019, the percentage of EU residents between 65 and 74 who had used the internet in the previous 3 months rose from 26% to 60%, while the number making internet purchases grew from 8% to 21% [26]. Eurostat does not publish EU-wide data for those seventy-five and above, but, by means of an example, in Spain, the frequency of internet use in the previous three months for this age group increased from 3% to 29% between 2010 and 2019, and online purchases rose from less than 1% to 5% [27]. The recent upward trends in population aging and in internet usage by older persons are also matched by overall rises in cyber fraud in several countries [20,28], which further highlights the need for increased research interest in fraud against senior citizens in digital society [29]. One issue to consider is that fraud is an extremely broad category, thus, to further understand of the problem and to design effective interventions to reduce the incidence and impact, we should aim to be as specific as possible. As Cross highlights “it may be important to note the different types of fraud that exist and how this may differ based on the variable of age” [30]. Cross cites studies by Deevy et al. [31] and Jorna [32] that focus on particular scam types such as telemarketing, investment, lottery, or computer support schemes and find that these can affect senior citizens at a greater rate. A study carried out during COVID-19 highlighted the relevance of tech support scams for older persons [33].

In addition to the prevalence of fraud and the typologies of fraud experienced by older persons, the impact of victimization has also been the subject of academic interest. In terms of financial impact and older citizens, findings are inconclusive. For instance, Payne found that losses suffered by older persons during the pandemic were greater than those of younger generations [33], while Reynolds found age to be relevant for banking fraud but not identity theft and credit card fraud [34]. Aside from economic consequences, fraud has been shown to have psychological effects, cause mental and physical health problems, damage a person’s reputation, and produce positive and negative behavior changes [35]. With respect to older citizens, research has shown that the health and well-being of individuals can be affected even when there has not been a financial loss [36]. A strong victim-blaming discourse has also been identified in relation to online fraud victimization of seniors, which can increase feelings of embarrassment or the negative impacts on well-being [37]. Nevertheless, despite the growing body of literature on the impacts of fraud on older adults, it was recently concluded that, with respect to many types of online victimization, “We know little about the prevalence and reporting rates of such harms among older citizens, nor about their financial, emotional, psychological, and other impacts” [38]. Moreover, there has been little attempt to compare the impacts between older and younger citizens, which could help guide interventions.

Thus, the present study aims to add to these debates by analyzing data from the European Commission Survey on Scams and Fraud Experienced by Consumers to examine whether older adults in Europe are more likely to suffer consumer fraud and to identify the types of fraud more likely to be suffered by older adults, as well as the financial and non-financial impacts in comparison to younger generations.

## 2. Materials and Methods

Based on the overview of the extant literature, the aforementioned aims were formulated as the following research questions:

1. In comparison to other age groups, are older persons less likely to suffer consumer fraud?
2. In comparison to other age groups, are older persons less likely to suffer consumer fraud via online channels?
3. In comparison to other age groups, what types of consumer fraud are older persons more likely to suffer?
4. In comparison to other age groups, does consumer fraud have a greater financial and non-financial impact on older persons?
5. In comparison to other age groups, does online consumer fraud have a greater financial and non-financial impact on older persons?

The data to respond to these questions come from the survey on “Scams and Fraud Experienced by Consumers” conducted by Ipsos on behalf of the European Commission in 2019 [39]. This survey was conducted in 2019 in the 28 EU Member States, as well as Iceland and Norway. For coherence with the original Commission report, the present study analyzes the data from respondents in the 28 EU Member States (*n* = 26,735). In each country, at least 1000 respondents aged 18 or over were interviewed using Computer-Assisted Telephone Interviewing (CATI), except Luxembourg, Malta, and Cyprus, which had a minimum sample size of 500 due to their smaller population. Stratified random sampling was employed in each country to obtain a sample that was representative in terms of age, gender, and phone ownership. To ensure representativity on these variables, the sample was weighted using a post-stratification weight that included age, gender, phone ownership, and a design weight. Population weighting was then implemented so that the weighted sample size was proportionate to the size of the eligible population. The population targets were based on Eurostat and Eurobarometer data. A least filled method was used with respondents who had experienced more than one type of fraud, meaning they were not asked about their last fraud experience, but about the fraud for which the administrators had the least answers at the time of the interview. Thus, to correct the proportions and profiles, a further weighting step was implemented for the subsample of fraud victims. The survey administrators made the weights available with the dataset, and the weighted samples are used in the analysis herein.

The outcome variable for RQ1 is whether the respondent had personally experienced any one of nine types of scams or fraud in the previous two years when purchasing goods or services either offline or online. As established in the survey questionnaire, the nine types of fraud are:

1. You ordered free or relatively cheap products or services, but it turned out you had been tricked into a costly monthly subscription.
2. You bought what you thought was a good deal, but you never received the goods/service or the goods/services turned out to be fake or non-existent.
3. You received a fake invoice for products that you had not ordered, and you were asked to pay the cost.
4. You were contacted—by phone, face to face, by email, or by another means—by someone pretending to be from a legitimate organization such as a bank, telephone or internet service provider, or government department and asked to provide (or confirm) personal information.
5. You were approached—by phone, face to face, by email, or by another means—or you accessed a website and were informed that you had a computer or internet problem. Then, you were asked for your personal details and your bank or credit card details to have the problem solved.
6. You were promised you would receive a good, a service, a rebate, or an important investment gain if you transferred or invested money.
7. You bought tickets for an event, concert, or travel, but it turned out the tickets were not genuine and/or you never received them.
8. You were contacted by someone pretending to be from a legitimate organization, such as a bank, internet provider, or government who claimed there were problems with your account or other documentation and threatened you with harm if you did not pay to resolve the problem.
9. You received notification of a lottery win or a competition win but were informed you would need to pay a fee or buy a product in order to collect your prize.

With respect to RQ2, the outcome variable is whether the respondent suffered any one of the previous scams or frauds and whether this was experienced via any of the following: email, mobile messaging channels such as WhatsApp or Facebook messenger, an online advertisement on a social media website, blog, or forum, or on a non-social media website.

The survey administrators (IPSOS-MORI) grouped the nine scam and fraud types enumerated above into three dummy-coded variables: buying scam (1–3), identity theft (4, 5), and monetary fraud (6–9). Thus, RQ3 has three outcome variables corresponding to whether the respondent has experienced fraud from each of the three categories.

With regard to RQ4 (any fraud) and RQ5 (online fraud), there are six outcome variables corresponding to different negative impacts suffered as a result of a scam or fraud. Firstly, the financial impact is measured by an ordinal variable consisting of five categories of total financial loss: €0, less than €50, more than €50 but less than €500, more than €500 but less than €2000, and more than €2000. To facilitate the analytic strategy, this was converted into a dichotomous variable for whether fraud victims suffered a financial loss or not. The non-financial negative impacts are measured by five binary variables for whether experiencing the fraud made the respondent angry, irritated, embarrassed, or stressed, or had a negative effect on their physical health.

For each research question, the main predictor variable of interest is the age of the victim, which is a categorical variable divided into those respondents who are 18–34, 35–50, 51–64, and 65 and above. Furthermore, when analyzing each question, we control for a number of other predictors based on the extant literature, namely gender, education (converted to low, medium, or high by survey administrators), financial situation (easy or difficult), if the respondent is the only adult in their household, if they live in a rural or urban area, internet use for private purposes (daily, weekly, less than weekly, never), online shopping, if they have seen any fraud awareness-raising adverts or campaigns, if they avoid clicking on suspicious links from unknown senders, if they install anti-spam or antivirus software in their devices, and if they check the credibility of vendors. The descriptive statistics of the predictor variables can be found in Table 1.

To answer the five questions, we begin with a descriptive overview with chi-squared tests to identify potential relationships between the age group variable and the outcome variables. Given that this initial descriptive overview does not take into account factors such as internet use, online shopping, or living alone, which may explain any different rates found with regard to older consumers, the main analytic strategy employed was generalized linear multilevel modelling. Multilevel modelling was used, as the rates of fraud victimization found by the survey differ between countries. For example, the countries with the highest rate of scams and fraud were Denmark, Ireland, and the United Kingdom, with 69, 68, and 67%, respectively, whereas the countries with the lowest prevalence were Hungary, Cyprus, and Bulgaria, with 28, 26, and 17%. As a consequence, it appears that individuals are nested in countries and are probably not independent. Multilevel modelling allows the clustered between-country differences to be accounted for in the analysis and is therefore preferable to multiple regression. All statistical analysis was conducted in R software [40] using the lme4 package [41].

## 3. Results

We will begin the results section with a descriptive overview of the relationship between age group and the outcome variables from our five research questions. Firstly, as can be seen in Table 2, 55.5% of respondents in the full weighted sample had experienced at least one form of consumer scam or fraud in the previous two years. However, we can see a certain amount of variation between age groups, in particular, the percentage of respondents aged 65 or above who experienced fraud is lower than the other age groups. Experiencing fraud through an online channel in the previous two years was reported by 31.9% of all respondents. The rate regarding older persons is 17.7%, which is less than half the online fraud rate found in the two youngest age groups. The chi-squared tests indicate that there is an association between age and fraud and age and online fraud victimization (*p* < 0.001 in both cases). However, it is important to note that this descriptive overview does not consider factors such as internet use and online shopping, which may explain the different rates found for older persons. This will be accounted for in Section 3.1 when we present the results of the multilevel models.

With respect to victimization by the different fraud types, Table 3 shows that the weighted rate of identity theft is 32.9%, monetary fraud is 38.8%, and buying scams is 22.6%. The age group patterns are similar to overall fraud victimization and online fraud victimization: the 35–49 age group experiences the highest prevalence in each fraud category while the 65+ age group has the lowest rate. However, in the case of identity theft, the difference is less than five percent.

Table 4 shows that, of those consumers in the sample who experienced a fraud or scam, most did not suffer any financial loss as a result. Moreover, most of those who did experience a financial impact lost less than €500, and only around 1% of victims lost more than €2000. With regard to older citizens, it appears the rates of small financial losses are lower than the other age groups, but the rates of higher financial losses are equal. The chi-square test finds a statistical difference between the reported values and the expected values in the data, however, the percentage differences between age groups are inconclusive.

The overall rates of financial losses for respondents who suffered fraud via an online channel are similar to the rates for fraud in general, as shown in Table 5. However, there are potentially some differences with respect to the age groups, since older adults have the highest rate of respondents who lost €50–499, the second highest rate for losses of €500–1999, and the highest rate for loss of over €2000, though the percentage differences in comparison to other age groups are inconclusive.

In terms of non-financial consequences from fraud and scams, the overall prevalences found in Table 6 are varied: the majority of victims reported feeling angry (55.6%) or irritated (68.5%), slightly less than a third felt stressed (30.4%), while 15.7% experienced embarrassment, and 6.2% suffered some form of negative effect on their physical health. The age group made up of people aged sixty-five and above seems to suffer non-financial impacts from fraud and scams at a greater rate than the others. This group reported the highest rates of anger, irritation, embarrassment, and negative physical consequences. The chi-square tests provide an indication of a statistical association between age group and these outcome variables, but not for stress.

Finally, the prevalence of all five types of reported non-financial consequences was slightly lower with regard to online consumer fraud and scams, as can be observed in Table 7. However, the pattern of greater non-financial impact for older persons is identified again and supported by the chi-square tests. In the case of online fraud, respondents aged sixty-five and over have reported the highest rate of three types of effects (anger, embarrassment, physical impact) and the second highest rate of the other two (irritation, stress).

### 3.1. Multilevel Modelling Results

Having provided a descriptive overview of the relationship between age and victimization and impact, we now proceed to model this relationship while accounting for the other predictor variables set out in the Materials and Methods section. We note that a small proportion of observations had to be deleted at this stage of the analysis due to missing data or answering “don’t know” or “other” to important questions such as internet use or gender. Given that deleted observations were less than two percent of the total sample, we are confident we can remove them without affecting the capacity of our analytic strategy to answer the research questions.

Firstly, Table 8 details the results for the multilevel model in which the outcome variables are whether the survey respondent reported having experienced any form of consumer fraud or scam in the previous two years or a consumer fraud or scam via online channels. As can be observed, when holding the other factors constant, the three age groups under sixty-five years old are less likely to suffer fraud via any channel than their older counterparts. The largest difference is with respect to the 18–34 age group, which is associated with thirty-four percent lower odds of experiencing fraud. This contrasts with the descriptive results that showed lower prevalence for the sixty-five and over group. It appears that this inversion of the direction of the effect is a consequence of controlling for internet use and online shopping, since the odds are greater than one or non-significant if we remove these two variables from the analysis. Table 8 also shows how the three younger age groups are more likely to suffer fraud via an online channel than older adults. We note that the sample used to analyze online fraud does not include respondents who never use the internet, which is more likely amongst older adults. These findings will be discussed in relation to routine activities theory in the discussion section.

In addition to the main predictor of interest, the model also estimates that other factors can explain the outcome variable of experiencing fraud. Notably, males are found to suffer consumer fraud offline and online at greater rates, and using the internet or shopping online more frequently are associated with increased likelihood of fraud in general and online fraud specifically. For instance, the odds of suffering consumer fraud are approximately sixty-five percent lower for those who never shop online in comparison to those who shop online every month. We do not find any consistent results for education, financial situation, or the variable for whether respondents live in a rural area, small town, or large town. The results for the protection measures are also inconsistent: not clicking on links, not using an antivirus, and not checking vendor identities appear to be associated with lower victimization, while reading terms and conditions is correlated with higher rates. We note that there was no issue with multicollinearity in terms of the variance inflation factor in these multilevel models, nor for all subsequent models presented in this section. The grouping variable for the multi-level analysis was the country variable, which has a moderate intraclass correlation coefficient of 0.05 in both models presented in Table 8.

Table 9 details the results for the multilevel models that estimate the correlation between identity theft, monetary fraud, and buying scams and the predictor variables. In various cases, age is again found to be associated with victimization. In the case of identity theft, respondents aged sixty-five and over are more likely to suffer this type of fraud. The greatest difference in odds is in comparison to those respondents aged 18–34, who are 40% less likely to report experiencing fraud. Older citizens are also associated with higher odds of monetary fraud in comparison to people aged 18–34, but no significant relationship is found with respect to the other age groups. On the other hand, the age group 35–49 is more likely to experience buying scams than the oldest group, though the findings are non-significant for people aged 18–34 and those aged 50–64. For all fraud categories, we find evidence that males are more likely victims and that more frequent internet activity positively predicts victimization. All three models estimate that respondents in an “easy” financial situation have lower odds of suffering fraud. The odds of the three types of fraud are also reduced for people who use the internet and shop online less frequently. The variables related to protection measures again show results that do not allow for clear conclusions to be drawn.

With respect to the financial impact of fraud, it appears that older persons who have experienced fraud are less likely to suffer financial consequences, as detailed in Table 10. The odds of suffering a monetary loss from fraud committed via any channel are 1.86 times greater for people in the 18–34 age group and 1.58 times greater with regard to that age group and online fraud. However, it should be noted that in order to estimate a multilevel model for the relationship between financial impact and age, it was necessary to convert the outcome variable to a dichotomous variable of financial impact (No/Yes). Unfortunately, this does not allow us to delve deeper into whether older citizens in Europe are more likely to suffer higher value losses, as indicated by the descriptive statistics.

Regarding demographic factors, higher education levels are correlated with suffering a financial impact, and, in terms of activities, we find using the internet less than daily is also associated with increased likelihood of financial losses. It also appears there may be a correlation between economic consequences and not avoiding suspicious links and only sometimes using an antivirus in comparison to always taking these precautionary measures.

Table 11 displays the results of the multilevel models with regard to the emotional, psychological, and physical effects of experiencing fraud. In this regard, we find strong evidence that younger people are less likely to suffer theses negative consequences than people aged sixty-five and over. Holding other factors constant, all three age groups suffer less anger, irritation, and embarrassment than the older persons, while the youngest are less likely to be stressed and the youngest two groups are significantly less likely to experience negative impacts on their physical health as a result of fraud victimization. Some of the reductions in odds are quite large; for example, the youngest age group is fifty-two percent less likely to be angry and forty-six percent less likely to suffer negative consequences to their physical health. The models also estimate that males are less likely to feel angry, irritated, stressed, or have negative physical effects than females, but the association regarding embarrassment is not significant. Furthermore, the financial situation of the victim appears to be particularly relevant, as those respondents who easily make ends meet every month suffer less non-financial impacts from fraud. The other factor for which there are clear consistent results is the financial impact of fraud, for which, as might be expected, victims who actually lose money have a higher likelihood of non-financial consequences. The intraclass correlation coefficient was low for some of these models, such as for physical effects (0.02).

In Table 12, we can see the results of the multilevel models with respect to the consequences of online consumer fraud. We again find strong indications of higher impact of fraud and scams for the oldest age group in comparison to the other three groups. The younger age groups are less likely to experience anger, irritation, embarrassment, stress, and negative physical effects than seniors. In the case of online fraud, males are less likely to feel angry, irritated, and stressed, but findings are not significant for embarrassment and physical consequences. Once more, respondents who consider their financial situation easier are associated with a lower likelihood of negative outcomes. Finally, experiencing a financial loss from the fraud is associated with a large positive effect size and, thus, it is a strong predictor of non-financial impact. The intraclass correlation coefficient was high for some of these models, for example, irritated (0.08).

## 4. Discussion

This study set out to respond to five questions regarding consumer fraud victimization of older citizens and its financial and non-financial impacts. In doing so, the findings have contributed to practical and theoretical discussions about economic crime against older adults in digital society.

Firstly, as noted in the introduction to this paper, there is a lack of consensus in prior research regarding the prevalence of financial victimization of older adults in comparison to other age groups [42], and our results add further fuel to this debate. On the one hand, it appears that older persons’ online routine activities may act as a protective factor for fraud since we found they experience lower overall consumer fraud prevalence rates as a result of lower internet use and online shopping. This is in line with previous research on consumer fraud from a routine activities perspective that highlights the relationship between purchasing behavior and consumer fraud [7,24]. However, when we hold constant the variables for frequency of internet use and online purchasing, senior citizens are found to be more likely to be victimized for fraud in general. This is relevant in the post-Covid world, as internet activities are continually increasing amongst older citizens, which means lower online routine activities may not be such a protective factor in the near future. Further research is needed to now update these findings after the digital acceleration induced by the pandemic. However, it should also be noted that, even when controlling for internet use and purchasing, younger people were still more likely to suffer online fraud. Thus, more extensive measurement and analysis of online routine activities is needed to understand predictive factors that may be associated with consumer fraud victimization, for example, related to risky online behaviors [43].

Secondly, frauds and scams with identity deception are found to be particularly relevant for older persons when holding other factors constant. That is, attacks where the offender pretends to be from a legitimate organization, such as a government institution, to obtain sensitive information from the victim, or “tech support scams”, where fraudsters pretend to be from a legitimate technology company and inform the victim that there is a problem with their device, but they can fix it for a fee. The odds ratios indicate substantive differences between the age groups and, moreover, similar results have been found in previous studies [33]. However, it should be noted that the basic percentage rates were inconclusive. It has been suggested that offenders specifically design these types of attacks for older victims, for example, to take advantage of insecurities regarding technology or a greater willingness to trust authorities [11]. This type of finding about specific fraud typologies can be informative for the design of awareness-raising and prevention campaigns targeted at senior citizens. The institutions responsible for programs that aim to protect older citizens should be aware of the most relevant threats they face and should monitor how these change over time. In this sense, our findings indicate that it is important to consider attacks that use a mix of vectors, for instance, landline telephones and computers or SMS and fraudulent websites. Similarly, when raising awareness about social engineering and how to respond if in doubt, programs for senior citizens should include material that enables them to check the veracity of people who contact them supposedly on behalf of recognized organizations. To ensure the effective dissemination of this information, prevention programs should make use of a diversity of channels to reach all segments of the sixty-five and above demographic group [8].

Finally, one of the most important conclusions of this study is that all non-financial consequences of fraud are greater for older persons, even though they are less likely to suffer an economic loss. This links to prior research on victim blaming and fear of incompetence in digital society. Previous studies have explored how older adults, even fraud victims themselves, often use labels such as greedy, gullible, or naïve to describe those that are victimized by fraudsters [37,44], and, in this sense, they attribute a certain degree of responsibility for their victimization. These discourses may exacerbate the feelings of anger or embarrassment that have been found herein. Moreover, research has shown that older persons sometimes fear that their family and friends will consider them incapable of managing their personal affairs in digital society after suffering fraud. For example, they worry that they will think they should not have full control of their own finances [9]. It is easy to imagine how these types of concerns can add to the negative emotional and psychological impacts of consumer fraud. Relatedly, older citizens are generally in worse physical health, so the emotional and psychological effects of fraud are more likely to develop into or worsen existing physical issues. This may explain why we find a greater likelihood of negative physical effects from fraud for those persons aged sixty-five or over.

A clear practical implication of our findings on the impact of consumer fraud and scams is that interventions with older fraud victims should not only focus on recovering funds but also on well-being and social connections. As Segal et al. state, “programs should include psychological guidance that would help older consumers deal with the emotional reactions that commonly follow consumer fraud” [44]. There are various examples of best practices of support services for fraud victims from around the world [9]. However, these are often specialized services, and it is unclear whether the needs of fraud victims are adequately considered in European countries such as Spain, where the authors of the present study are based and where the response mainly corresponds to police forces or financial institutions. Research suggests that fraud victims are often dissatisfied with the response they receive from the police or banking and insurance companies [45], and the importance of training professionals who work with older persons has been noted [44]. Unfortunately, the issue of responses to fraud has only been examined in a very limited number of countries. Given the extent of fraud victimization in digital society, it seems pertinent for European countries to also explore how responses to fraud are experienced by victims and how responses can be improved.

## 5. Conclusions and Limitations

This study finds that the sixty-five and over age group suffers consumer fraud at a lower percentage rate than younger age groups. This finding is the same for all fraud, fraud that occurs via online channels, as well as identity theft, monetary fraud, and buying scams. However, when holding constant frequency of internet use and online purchasing, older citizens are found to have greater odds of experiencing consumer fraud in general and identity fraud, but lower odds of fraud via online channels.

We also find that, while older persons are less likely to suffer a financial impact from consumer fraud victimization, they are more likely to suffer all non-financial impacts measured in this study, such as anger, embarrassment, and negative effects on their physical health.

As with all studies based on observational data obtained via cross-sectional surveys, there are limitations that are both relevant to interpreting the results and promoting future research on the topic. For instance, the inconsistencies found with regard to protective measures may be due to these variables being related to using the internet less frequently, or it may be that people who do these things at a greater rate are more likely to detect fraudulent attempts than those who do not. In any case, it underlines the difficulty of using cross-sectional survey data to evaluate the efficacy of cybercrime prevention mechanisms, as found in prior research [46]. To further the understanding of fraud against older persons, future studies should aim to analyze individual countries in greater depth with larger sample sizes and with the inclusion of a wider range of questions on online routine activities and the consequences of victimization. An important area of future research is the positive and negative individual behavioral responses of older citizens to victimization in digital society and how these impact digitally active citizens.

## Figures and Tables

**Table 1:** Predictor variables in weighted sample.

Variable	Respondents	%
Age		
18–34	6748	25.2
35–49	6674	25.0
50–64	7016	26.2
65+	6297	23.6
Gender		
Female	13,814	51.7
Male	12,917	48.3
Education		
Low	2421	9.2
Medium	11,333	43.2
High	12,453	47.5
Financial situation		
Difficult	8720	32.6
Easy	16,893	63.2
Don’t know	1123	4.2
Live only adult		
Yes	6667	24.9
No	20,068	75.1
Area they live		
Rural	7400	27.8
Small or medium town	10,172	38.2
Large town	9088	34.0
Internet use		
Every day	18,873	70.7
Every week	3205	12.0
Less than every week	2375	8.9
Never	2231	8.4
Online shopping		
Every month	12,083	45.3
Less than every month	9849	36.9
Never	4737	17.8
Seen fraud awareness		
Yes	17,968	67.2
No	8767	32.8
Avoid clicking suspicious links		
Always	18,575	69.5
Sometimes	3390	12.7
Never	4770	17.8
Use antivirus		
Always	16,116	60.3
Sometimes	3435	12.8
Never	7184	26.9
Check credibility of vendor		
Always	13,398	50.1
Sometimes	7576	28.3
Never	5761	21.6

**Table 2:** Percentage of consumer fraud and online fraud victimization in weighted sample.

Age Group	% Fraud Victimization	% Online Fraud Victimization
Overall	55.5	31.9
18–34	57.2	38.5
35–49	61.8	40.3
50–64	55.7	30.5
65+	46.7	17.7
Chi-square test	*p* < 0.001	*p* < 0.001

**Table 3:** Percentage of victimization of different fraud types in weighted sample.

Age Group	% Identity Theft Victimization	% Monetary Fraud Victimization	% Buying Scam
Overall	32.9	38.8	22.6
18–34	32.1	39.1	26.1
35–49	37.2	45.0	28.6
50–64	33.5	40.0	21.3
65+	28.5	30.5	13.7
Chi-square test	*p* < 0.001	*p* < 0.001	*p* < 0.001

**Table 4:** Percentage of financial losses in weighted sample.

Age Group	% 0€	% < €50	% €50–499	% €500–1999	% €2000+
Overall	75.7	10.5	9.6	2.3	0.9
18–34	71.8	14.2	10.5	2.1	0.6
35–49	75.9	10.4	9.5	2.4	1.0
50–64	76.5	8.9	9.8	2.5	1.1
65+	80.0	7.4	7.9	2.3	1.1
Chi-square test: *p* < 0.001					

**Table 5:** Percentage of financial losses from online fraud in weighted sample.

Age Group	% 0€	% < €50	% 50–499	% 500–1999	% 2000+
Overall	75.0	11.0	10.5	2.2	0.7
18–34	71.4	14.8	10.6	2.5	0.3
35–49	77.2	9.9	9.2	2.1	0.7
50–64	75.9	9.7	11.1	1.8	0.9
65+	76.1	7.5	12.2	2.4	1.2
Chi-square test: *p* < 0.001					

**Table 6:** Percentage of non-financial impact in weighted sample.

Age Group	% Angry	% Irritated	% Embarrassed	% Stressed	% Physical Impact
Overall	55.6	68.5	15.7	30.4	6.2
18–34	50.1	64.2	15.0	30.1	4.9
35–49	55.1	67.1	15.1	29.8	5.8
50–64	56.1	71.6	15.5	31.4	6.8
65+	63.3	72.2	18.0	30.2	8.1
Chi-square test	*p* < 0.001	*p* < 0.001	*p* < 0.05	*p* = 0.43	*p* < 0.001

**Table 7:** Percentage of non-financial impact from online fraud in weighted sample.

Age Group	% Angry	% Irritated	% Embarrassed	% Stressed	% Physical Impact
Overall	50.5	65.0	15.0	27.6	4.5
18–34	46.4	60.9	13.5	28.0	2.8
35–49	51.3	64.4	14.9	26.5	5.3
50–64	51.3	68.9	15.7	28.7	4.5
65+	57.5	68.4	17.6	27.5	6.6
Chi-square test	*p* < 0.001	*p* < 0.001	*p* < 0.05	*p* = 0.47	*p* < 0.001

**Table 8:** Odds ratios and 95% CI for fraud and online fraud victimization.

Variable	Fraud(*n* = 26,225)	Online Fraud (*n* = 23,807)
Age (ref = 65+)		
18–34	0.66 (0.60–0.72) ***	1.22 (1.13–1.38) ***
35–49	0.84 (0.77–0.93) ***	1.36 (1.26–1.55) ***
50–64	0.86 (0.79–0.94) ***	1.16 (1.08–1.32) ***
Gender (ref = female)		
Male	1.18 (1.11–1.24) ***	1.27 (1.20–1.35) ***
Education (ref = low)		
Medium	0.96 (0.86–1.06)	0.99 (0.87–1.13)
High	1.11 (1.00–1.24)	1.17 (1.03–1.33) *
Financial situation		
(ref = difficult)		
Easy	0.87 (0.82–0.93) ***	0.94 (0.88–1.00)
Live only adult (ref = yes)		
No	1.00 (0.94–1.07)	0.91 (0.85–0.98) **
Area live (ref = rural)		
Small/medium town	0.93 (0.87–1.00)	1.00 (0.92–1.07)
Large town	1.09 (1.01–1.17) *	1.09 (1.01–1.18) *
Internet use		
(ref = every day)		
Every week	0.81 (0.74–0.89) ***	0.71 *** (0.64–0.78)
Less than every week	0.89 (0.80–0.99) *	0.66 *** (0.58–0.75)
Never	0.87 (0.77–1.03)	NA
Online shopping		
(ref = every month)		
Less than every month	0.73 (0.69–0.78) ***	0.73 (0.68–0.77) ***
Never	0.37 (0.33–0.41) ***	0.32 (0.27–0.37) ***
Seen fraud awareness campaign (ref = yes)		
No	0.96 (0.90–1.02)	1.03 (0.96–1.10)
Avoid clicking suspicious links (ref = always)		
Sometimes	1.04 (0.95–1.13)	1.05 (0.96–1.15)
Never	0.70 (0.66–78) ***	0.61 (0.55–0.68) ***
Use antivirus (ref = always)		
Sometimes	0.80 (0.74–0.88) ***	0.80 (0.73–0.88) ***
Never	0.69 (0.64–0.74) ***	0.73 (0.67–0.79) ***
Check credibility of vendor (ref = always)		
Sometimes	0.96 (0.90–1.02)	0.97 (0.90–1.03)
Never	0.72 (0.66–0.78) ***	0.82 (0.82–0.75) ***
Read terms and conditions (ref = always)		
Sometimes	1.05 (0.99–1.13)	1.15 (1.07–1.24) ***
Never	1.30 (1.20–1.41) ***	1.47 (1.35–1.61) ***

*** *p* < 0.001, ** *p* < 0.01, * *p* < 0.05. NA: Not Applicable.

**Table 9:** Odds ratio and 95% CI for identity theft, monetary fraud, and buy scams.

Variable	Identity Theft (*n* = 26,225)	Monetary Fraud (*n* = 26,225)	Buying Scams (*n* = 26,225)
Age (ref = 65+)			
18–34	0.60 (0.55–0.66) ***	0.76 (0.69–0.84) ***	1.07 (0.96–1.20)
35–49	0.81 (0.73–0.89) ***	1.04 (0.95–1.14)	1.37 (1.23–1.52) ***
50–64	0.81 (0.74–0.89) ***	1.01 (0.92–1.10)	1.08 (0.97–1.20)
Gender (ref = female)			
Male	1.18 (1.11–1.25) ***	1.22 (1.15–1.29) ***	1.23 (1.15–1.31) ***
Education (ref = low)			
Medium	1.09 (0.97–1.22)	1.03 (0.92–1.15)	0.91 (0.80–1.04)
High	1.15 (1.02–1.29) *	1.25 (1.11–1.39) ***	1.07 (0.94–1.22)
Financial situation			
(ref = difficult)			
Easy	0.93 (0.87–0.99) *	0.85 (0.79–0.91) ***	0.77 (0.71–0.82) ***
Live only adult (ref = yes)			
No	1.04 (0.97–1.12)	0.94 (0.88–1.00)	0.99 (0.92–1.07)
Area live (ref = rural)			
Small/medium town	0.98 (0.91–1.05)	0.85 (0.79–0.91) ***	0.92 (0.84–0.99) *
Large town	1.01 (0.93–1.09)	0.99 (0.92–1.07)	1.06 (0.97–1.15)
Internet use			
(ref = every day)			
Every week	0.85 (0.77–0.94) ***	0.79 (0.72–0.87) ***	0.72 (0.64–0.80) ***
Less than every week	0.90 (0.80–1.01)	0.88 (0.79–0.98) *	0.87 (0.76–1.00)
Never	0.82 (0.69–0.98) *	0.85 (0.72–1.00)	0.74 (0.58–0.94) *
Online shopping			
(ref = every month)			
Less than every month	0.73 (0.68–0.78) ***	0.82 (0.77–0.87) ***	0.74 (0.68–0.79) ***
Never	0.42 (0.37–0.48) ***	0.42 (0.37–0.47) ***	0.28 (0.24–0.34) ***
Seen fraud awareness campaign (ref = yes)			
No	1.00 (0.93–1.06)	0.98 (0.92–1.04)	1.01 (0.94–1.08)
Avoid clicking suspicious links (ref = always)			
Sometimes	1.08 (0.99–1.19)	1.14 (1.05–1.24) ***	1.25 (1.14–1.38) ***
Never	0.81 (0.74–0.90) ***	0.83 (0.76–0.91) ***	0.89 (0.79–0.99) *
Use antivirus (ref = always)			
Sometimes	0.88 (0.81–0.97) *	0.90 (0.82–0.98) *	1.10 (1.00–1.21)
Never	0.74 (0.65–0.77) ***	0.73 (0.68–0.79) ***	0.91 (0.83–1.00)
Check credibility of vendor (ref = always)			
Sometimes	0.87 (0.81–0.93) ***	0.96 (0.90–1.02)	1.05 (0.98–1.13)
Never	0.73 (0.67–0.80) ***	0.81 (0.75–0.88) ***	0.88 (0.80–0.97) *
Read terms and conditions (ref = always)			
Sometimes	1.02 (0.95–1.09)	0.99 (0.93–1.06)	0.98 (0.91–1.06)
Never	1.13 (1.04–1.22) *	1.09 (1.01–1.18) *	1.26 (1.15–1.38) ***

*** *p* < 0.001, * *p* < 0.05.

**Table 10:** Odds ratios and 95% CI for financial impact from fraud and online fraud victimization.

Variable	Fraud (*n* = 12,519)	Online Fraud (*n* = 7061)
Age (ref = 65+)		
18–34	1.86 (1.60–2.16) ***	1.58 (1.29–1.93) ***
35–49	1.45 (1.25–1.68) ***	1.09 (0.90–1.35)
50–64	1.35 (1.17–1.57) ***	1.12 (0.92–1.38)
Gender (ref = female)		
Male	1.00 (0.92–1.09)	1.03 (0.92–1.16)
Education (ref = low)		
Medium	1.24 (1.02–1.47) *	1.57 (1.21–2.03) ***
High	1.15 (0.96–1.37)	1.32 (1.02–1.71) *
Financial situation		
(ref = difficult)		
Easy	0.84 (0.76–0.92) ***	1.04 (0.92–1.18)
Live only adult (ref = yes)		
No	0.97 (0.87–1.07)	0.90 (0.79–1.03)
Area live (ref = rural)		
Small/medium town	0.99 (0.89–1.10)	0.89 (0.77–1.02)
Large town	1.07 (0.95–1.19)	0.94 (0.81–1.08)
Internet use		
(ref = every day)		
Every week	1.18 (1.03–1.36) *	1.19 (0.98–1.44)
Less than every week	1.55 (1.31–1.84) ***	1.84 (1.46–2.32) ***
Never	0.93 (0.67–1.28)	NA
Online shopping		
(ref = every month)		
Less than every month	1.08 (0.98–1.19)	1.16 (1.03–1.32) *
Never	0.74 (0.59–0.90) ***	0.90 (0.64–1.21)
Seen fraud awareness campaign (ref = yes)		
No	0.96 (0.87–1.05)	1.12 (0.99–1.27)
Avoid clicking suspicious links (ref = always)		
Sometimes	1.11 (0.98–1.26)	0.97 (0.82–1.14)
Never	1.49 (1.29–1.71) ***	1.54 (1.26–1.88) ***
Use antivirus (ref = always)		
Sometimes	1.24 (1.09–1.41) ***	1.39 (1.17–1.65) ***
Never	0.95 (0.84–1.08)	1.01 (0.86–1.20)
Check credibility of vendor (ref = always)		
Sometimes	1.06 (0.97–1.17)	1.03 (0.91–1.17)
Never	0.96 (0.85–1.11)	0.90 (0.76–1.07)
Read terms and conditions (ref = always)		
Sometimes	0.74 (0.67–0.82) ***	0.63 (0.55–0.72) ***
Never	0.89 (0.79–1.00)	0.72 (0.62–0.84) ***

*** *p* < 0.001, * *p* < 0.05. NA: Not Applicable.

**Table 11:** Odds ratios and 95% CI for non-financial effects of fraud (*n* = 12,519).

Variable	Angry	Irritated	Embarrassment	Stressed	Physical Effect
Age (ref = 65+)					
18–34	0.48 (0.42–0.55) ***	0.57 (0.50–0.66) ***	0.71 (0.59–0.84) ***	0.85 (0.74–0.98) *	0.54 (0.42–0.71) ***
35–49	0.60 (0.53–0.68) ***	0.68 (0.59–0.78) ***	0.80 (0.67–0.94) ***	0.89 (0.78–1.01)	0.77 (0.61–0.99) *
50–64	0.67 (0.60–0.76) ***	0.81 (0.71–0.93) ***	0.83 (0.71–0.98) *	0.88 (0.77–1.00)	0.79 (0.64–1.02)
Gender (ref = female)					
Male	0.63 (0.58–0.68) ***	0.62 (0.57–0.67) ***	0.92 (0.83–1.02)	0.68 (0.63–0.74) ***	0.85 (0.73–0.99) *
Education (ref = low)					
Medium	1.16 (0.99–1.35)	1.06 (0.90–1.24)	1.05 (0.84–1.29)	0.84 (0.72–0.99) *	0.58 (0.45–0.74) ***
High	1.01 (0.87–1.18)	1.05 (0.89–1.24)	1.01 (0.81–1.24)	0.87 (0.74–1.03)	0.53 (0.41–0.68) ***
Financial situation					
(ref = difficult)					
Easy	0.72 (0.66–0.79) ***	0.79 (0.72–0.87) ***	0.75 (0.67–0.8) ***	0.73 (0.67–0.80) ***	0.41 (0.35–0.49) ***
Live only adult (ref = yes)					
No	1.10 (1.00–1.20)	1.03 (0.94–1.14)	0.92 (0.81–1.04)	0.93 (0.84–1.02)	0.85 (0.72–1.02)
Area live (ref = rural)					
Small/medium town	0.85 (0.77–0.94) ***	0.96 (0.87–1.06)	1.14 (1.00–1.30) *	1.00 (0.90–1.11)	0.75 (0.61–0.90) ***
Large town	0.82 (0.74–0.91) ***	0.87 (0.79–0.97) *	0.97 (0.84–1.11)	0.87 (0.78–0.96) *	0.86 (0.70–1.04)
Internet use					
(ref = every day)					
Every week	0.82 (0.73–0.94) ***	0.86 (0.75–0.98) *	1.29 (1.10–1.52) ***	0.97 (0.85–1.11)	1.05 (0.82–1.34)
Less than every week	1.21 (1.03–1.43) *	0.88 (0.75–1.05)	0.93 (0.74–1.12)	0.98 (0.83–1.16)	1.42 (1.07–1.83) ***
Never	1.61 (1.13–1.91) ***	1.26 (0.97–1.69)	1.03 (0.68–1.33)	0.66 (0.50–0.88) ***	1.05 (0.62–1.47)
Online shopping					
(ref = every month)					
Less than every month	0.91 (0.83–0.99) *	0.97 (0.87–1.04)	0.89 (0.79–1.00) *	1.01 (0.92–1.11)	1.18 (0.99–1.41)
Never	0.77 (0.65–0.92) ***	0.81 (0.68–0.97) *	1.35 (1.08–1.69) **	0.83 (0.69–1.00)	1.17 (0.85–1.61)
Seen fraud awareness campaign (ref = yes)					
No	0.90 (0.83–0.98) *	0.89 (0.82–0.98) *	0.94 (0.84–1.05)	0.89 (0.82–0.97) *	0.93 (0.78–1.10)
Avoid clicking suspicious links (ref = always)					
Sometimes	0.96 (0.85–1.07)	1.04 (0.92–1.17)	1.19 (1.02–1.38) *	1.23 (1.09–1.39) ***	1.44 (1.15–1.79) ***
Never	0.94 (0.79–1.04)	0.91 (0.80–1.06)	1.41 (1.16–1.61) ***	1.44 (1.26–1.65) ***	1.34 (1.06–1.69) *
Use antivirus (ref = always)					
Sometimes	1.06 (0.93–1.19)	1.03 (0.90–1.16)	1.35 (1.16–1.57) ***	1.35 (1.19–1.53) ***	0.99 (0.77–1.26)
Never	0.96 (0.87–1.09)	0.84 (0.75–0.95) ***	1.04 (0.89–1.20)	0.81 (0.73–0.94) ***	1.13 (0.88–1.36)
Check credibility of vendor (ref = always)					
Sometimes	0.94 (0.86–1.02)	0.98 (0.90–1.08)	1.14 (1.01–1.28) *	0.96 (0.87–1.05)	0.93 (0.77–1.11)
Never	0.83 (0.74–0.93) ***	0.91 (0.81–1.04)	1.05 (0.90–1.23)	1.16 (1.02–1.31) *	1.00 (0.80–1.26)
Read terms and conditions (ref = always)					
Sometimes	1.01 (0.92–1.11)	1.25 (1.13–1.38) ***	0.95 (0.84–1.08)	0.92 (0.84–1.02)	0.63 (0.52–0.75) ***
Never	0.93 (0.83–1.03)	0.94 (0.83–1.05)	0.90 (0.77–1.04)	0.75 (0.67–0.84) ***	0.79 (0.64–0.97) *
Financial loss (ref = no)					
Yes	5.10 (4.57–5.65) ***	3.72 (3.32–4.16) ***	5.62 (5.06–6.26) ***	3.96 (3.62–4.33) ***	4.90 (4.18–5.74) ***

*** *p* < 0.001, ** *p* < 0.01, * *p* < 0.05.

**Table 12:** Odds ratios and 95% CI for non-financial effects of online fraud (*n* = 7061).

Variable	Angry	Irritated	Embarrassment	Stressed	Physical Effect
Age (ref = 65+)					
18–34	0.48 (0.40–0.58) ***	0.56 (0.46–0.67) ***	0.65 (0.50–0.83) ***	0.79 (0.65–0.96) *	0.35 (0.23–0.55) ***
35–49	0.62 (0.51–0.74) ***	0.70 (0.58–0.84) ***	0.87 (0.69–1.11)	0.81 (0.66–0.98) *	0.93 (0.64–1.37)
50–64	0.69 (0.57–0.82) ***	0.86 (0.72–1.04)	0.94 (0.74–1.20)	0.84 (0.69–1.03)	0.68 (0.46–0.98) *
Gender (ref = female)					
Male	0.63 (0.57–0.70) ***	0.65 (0.58–0.72) ***	0.92 (0.80–1.06)	0.70 (0.63–0.79) ***	0.87 (0.70–1.13)
Education (ref = low)					
Medium	1.31 (1.05–1.64) *	1.29 (1.04–1.62) *	1.10 (0.80–1.51)	1.02 (0.80–1.30)	0.44 (0.30–0.67) ***
High	1.14 (0.91–1.42)	1.09 (1.09–1.70) *	0.95 (0.69–1.31)	1.02 (0.81–1.29)	0.41 (0.27–0.61) ***
Financial situation					
(ref = difficult)					
Easy	0.69 (0.62–0.78) ***	0.78 (0.69–0.88) ***	0.81 (0.70–0.95) *	0.73 (0.65–0.82) ***	0.42 (0.33–0.54) ***
Live only adult (ref = yes)					
No	1.06 (0.94–1.20)	1.09 (0.96–1.23)	0.93 (0.78–1.09)	0.98 (0.86–1.12)	0.85 (0.61–1.04)
Area live (ref = rural)					
Small/medium town	1.04 (0.91–1.18)	1.20 * (1.05–1.37)	1.17 (0.98–1.40)	1.09 (0.95–1.25)	0.72 (0.53–0.98) *
Large town	0.94 (0.82–1.08)	1.02 (0.89–1.16)	1.07 (0.88–1.28)	1.05 (0.91–1.21)	0.94 (0.70–1.26)
Internet use					
(ref = every day)		0.60 (0.50–0.72) ***	1.29 (1.08–1.69) ***	0.72 (0.59–0.89) ***	0.68 (0.44–1.06)
Every week	0.69 (0.58–0.83) ***	1.04 (0.81–1.34)	0.63 (0.45–0.88) *	1.08 (0.85–1.38)	0.71 (0.44–1.15)
Less than every week	1.58 (1.22–2.03) ***				
Online shopping					
(ref = every month)					
Less than every month	0.77 (0.69–0.86) ***	1.06 (0.94–1.19)	0.88 (0.67–0.91) ***	0.93 (0.83–1.06)	1.12 (0.85–1.46)
Never	0.73 (0.55–0.97) *	0.76 (0.57–1.01)	1.01 (0.68–1.50)	0.77 (0.57–1.06)	2.44 (1.47–4.04)
Seen fraud awareness campaign (ref = yes)					
No	0.79 (0.71–0.89) ***	0.92 (0.82–1.03)	1.15 (0.98–1.34)	0.88 (0.78–1.00)	1.00 (0.76–1.31)
Avoid clicking suspicious links (ref = always)					
Sometimes	1.03 (0.89–1.20)	0.96 (0.83–1.12)	1.43 (1.18–1.73) ***	1.14 (0.97–1.34)	1.13 (0.80–1.60)
Never	0.87 (0.71–1.06)	0.74 (0.60–0.91) ***	1.68 (1.32–2.13) ***	1.36 (1.11–1.68) ***	1.66 (1.13–2.44) ***
Use antivirus (ref = always)					
Sometimes	1.06 (0.83–1.15)	0.86 (0.73–1.01)	0.95 (0.77–1.18)	1.00 (0.84–1.18)	0.74 (0.50–1.10)
Never	0.80 (0.69–0.94) ***	0.94 (0.80–1.09)	0.73 (0.58–0.90) ***	0.75 (0.63–0.89) ***	0.98 (0.70–1.39)
Check credibility of vendor (ref = always)					
Sometimes	0.94 (0.84–1.05)	1.10 (0.98–1.24)	1.14 (1.03–1.42) *	0.95 (0.84–1.08)	1.07 (0.81–1.42)
Never	0.83 (0.71–0.97) *	0.84 (0.72–0.99) *	1.23 (0.99–1.52)	0.95 (0.79–1.13)	1.44 (1.00–2.06)
Read terms and conditions (ref = always)					
Sometimes	0.99 (0.87–1.13)	1.18 (1.03–1.34) *	0.76 (0.64–0.90) ***	0.85 (0.74–0.97) *	0.66 (0.49–0.89) ***
Never	0.89 (0.77–1.04)	0.84 (0.72–0.97)	0.86 (0.71–1.05)	0.76 (0.65–0.89) ***	0.92 (0.66–1.27)
Financial loss (ref = no)					
Yes	5.89 (5.15–6.74) ***	4.94 (4.25–5.74) ***	5.91 (5.13–6.82) ***	4.28 (3.80–4.82) ***	5.66 (4.42–7.25) ***

*** *p* < 0.001, ** *p* < 0.01, * *p* < 0.05.

## Data Availability

Data are available on request.
